# Supplementary material for: Unifying the mechanism of mitotic exit control in a spatiotemporal logical model
Source: PLoS Biol. 2020 Nov 12;18(11):e3000917. doi: 10.1371/journal.pbio.3000917 (PMC7685450; doi:10.1371/journal.pbio.3000917)
Supplement: S1 Table — (PDF) [file pbio.3000917.s011.pdf]

Parameter	Definition	Value
ρ	Standard rate of reactions in Model 6	0.84
ρ_{slow}	Rate of Tem1 activation in absence of Lte1	0.012
ρ_{fast}	Rate of Tem1 activation in presence of Lte1	1
σ	Rate constant of spindle alignment	0.14
θ	Half-angular bud width	0.3

Table S1: Parameters used to simulate SPoC compentence.
