# Supplementary material for: Unifying the mechanism of mitotic exit control in a spatiotemporal logical model
Source: PLoS Biol. 2020 Nov 12;18(11):e3000917. doi: 10.1371/journal.pbio.3000917 (PMC7685450; doi:10.1371/journal.pbio.3000917)
Supplement: S2 Table — (PDF) [file pbio.3000917.s012.pdf]

Plasmid Name	Genotype	Type	Selection	Source
pX29	-	CEN	Leu/Amp	Reid et al. [2011]
pX78	*CDC5*	2μm	Leu/Amp	Caydasi et al. [2017]
pX79	*MOB1*	2μm	Leu/Amp	Caydasi et al. [2017]
pX80	*MOB1*	CEN	Ura/Amp	Caydasi et al. [2017]
pHT790	*MET3p-GBP-RFP*	CEN	Leu/Met/Amp	this study
pHT795	*MET3p-CLB2-CDC28-GBP-RFP*	CEN	Leu/Met/Amp	this study

Table S2: Plasmids used in this study.
