# Supplementary material for: Unifying the mechanism of mitotic exit control in a spatiotemporal logical model
Source: PLoS Biol. 2020 Nov 12;18(11):e3000917. doi: 10.1371/journal.pbio.3000917 (PMC7685450; doi:10.1371/journal.pbio.3000917)
Supplement: S3 Table — (PDF) [file pbio.3000917.s013.pdf]

Strain name	Genotype	Source
CKY329-3	*mob1*Δ*::klTRP1*	Caydasi et al. [2017]
T744	*CDC14-CFP::shHPH HIS3p-mRuby2-TUB1+3'UTR::URA3*	this study
pT747	*CDC14-CFP::shHPH HIS3p-mRuby2-TUB1+3'UTR::URA3 spo12*Δ*::KAN*	this study
E438	-	Brachmann et al. [1998]
T724	*CDC15-YFP::KAN*	this study
T725	*TEM1-YFP*	this study
Nud1-GFP	*Nud1-GFP::HIS3*	Huh et al. [2003]
pHT795	*Nud1-GFP::HIS3 bfa1*Δ*::KAN*	this study

Table S3: Strains used in this study.
