# Supplementary material for: Unifying the mechanism of mitotic exit control in a spatiotemporal logical model
Source: PLoS Biol. 2020 Nov 12;18(11):e3000917. doi: 10.1371/journal.pbio.3000917 (PMC7685450; doi:10.1371/journal.pbio.3000917)
Supplement: S1 Text — List of abbreviations, detailed descriptions of the FEAR network and MEN, and explanation of the scope of the model. (PDF) [file pbio.3000917.s027.pdf]

Supplementary Text S1

Abbreviations

- APC - Anaphase Promoting Complex
- ASC - Anaphase Specific Component
- CDK - Cyclin-Dependent Kinase
- CV - Coeffecient of Variation
- dSPB - daughter Spindle Pole Body
- DIC - Differential Interference Contrast
- FEAR - CdcFourteen Early Anaphase Release
- GAP - GTPase Activating Protein
- GBP - GFP-binding protein
- GEF - Guanine nucleotide Exchange Factor
- MEN - Mitotic Exit Network
- mSPB - mother Spindle Pole Body
- NLS - Nuclear Localization Signal
- ODE - Ordinary Differential Equations
- OE - OverExpression
- PDF - Probability Distribution Function
- PKN - Prior Knowledge Network
- SAC - Spindle Assembly Checkpoint
- SIN - Septation Initiation Network
- SPB - Spindle Pole Body
- SPoC - Spindle Position Checkpoint

The FEAR Network

The FEAR network is centred on phosphorylation of Net1, which leads to disassociation of Cdc14 from Net1 (Rock and Amon [2009]) (Figure ??). Net1 is phosphorylated by CDK, Cdc5 and Mob1-Dbf2 and some of this phosphorylation is reversed by PP2A-Cdc55 (Shou et al. [2002], Azzam et al. [2004], Ptacek et al. [2005], Queralt et al. [2006]). The FEAR signal is initiated by release of separase (Esp1) caused by destruction of securin (Pds1) by APC-Cdc20 upon release of the SAC. Esp1 functions in a complex with Slk19 and in conjunction with Zds1/2 to downregulate PP2A-Cdc55 and exclude it from the nucleus (Queralt and Uhlmann [2008], Rossio and Yoshida [2011]). This shifts the kinase/phosphatase balance in the nucleus to allow the hyper-phosphorylation of Net1 by CDK and Cdc5 that is required for FEAR. Further FEAR components are the nucleolar proteins Spo12 and Fob1, which participate through a poorly understood mechanism (Stegmeier et al. [2004], Tomson et al. [2009]). Condensation of the rDNA is also important for FEAR, mutants of the Hit1-Rsa1 snoRNP complex fail release Cdc14 in early anaphase (de los Santos-Velázquez et al. [2017]). There is some discussion whether Cdc14 is limited to the nucleus in early anaphase (Yellman and Roeder [2015]). Certainly it is not detectable by fluorescence microscopy outside of the nucleus at this stage, however the genetic interactions between FEAR and MEN components (Jaspersen et al. [1998]) suggest that it may be present in the cytoplasm at low levels, where it dephosphorylates cytoplasmic MEN components.

The MEN

Activation of the MEN is controlled by the SPoC which targets the Tem1 GTPase. Tem1 is regulated by the Bub2-Bfa1 complex, which acts as a GTPase-Activating Protein (GAP), forcing Tem1 into its inactive GDP-bound state (Pereira et al. [2000]). Bub2-Bfa1 localises asymmetrically to the Spindle Pole Bodies (SPBs), with a preference for the SPB that is closest to the bud (the daughter-bound SPB or dSPB, as opposed to the mother-bound SPB or mSPB). Tem1 also localises to the SPBs, primarily through interaction with Bub2-Bfa1, although, to a lesser extent, independently (Pereira et al. [2002], Caydasi et al. [2012]). Bub2-Bfa1's GAP activity can be inhibited by phosphorylation of Bfa1 by Cdc5 (Geymonat et al. [2003]), which also resides at the SPBs (Botchkarev and Haber [2017]). Bub2-Bfa1 localization is regulated by Kin4, which kinase protects Bfa1 by disrupting its stable localization at the SPB, keeping it away from Cdc5 (Caydasi and Pereira [2009]). This means that in the mother compartment, where Kin4 resides, Bfa1 is kept active (Maekawa et al. [2007]). When the dSPB enters the bud, Bfa1 is no longer protected by Kin4, allowing Cdc5 phosphorylation to occur. Kin4 itself is excluded from the bud compartment by the bud-localised protein Lte1 (Bertazzi et al. [2011], Falk et al. [2011]) and is activated by phosphorylation by Elm1 kinase (Caydasi et al. [2010]). Aside from regulation of Kin4, Lte1 has additional MEN-promoting activity, possibly acting through Bfa1 (Caydasi et al. [2017]). Lte1 has homology with other GEFs, and it was this acitvity which was thought to oppose

the GAP activity of Bub2-Bfa1 towards Tem1 (Bardin and Amon [2001]), however no GEF activity towards Tem1 was detected *in vitro* (Geymonat et al. [2009]). Upon entry of the dSPB into the bud, the Bub2-Bfa1 complex becomes inactive, allowing Tem1 to recruit the kinase Cdc15 to the SPB (Rock and Amon [2011]), which in turn recruits the Dbf2-Mob1 complex (Rock et al. [2013]). There Cdc15 phosphorylates Dbf2 (Mah et al. [2001]), activating the Dbf2-Mob1 complex which in turn leads to the full release of Cdc14. The exact details of the mechanism by which Mob1-Dbf2 promotes mitotic exit are not yet understood, however it is known that the complex enters the nucleus (Stoepel et al. [2005]), phosphorylates Net1 (Ptacek et al. [2005]) and phosphorylates Cdc14 near to its NLS, allowing Cdc14 to leave the nucleus (Mohl et al. [2009]).

Most of the MEN proteins localise to the SPB through interaction with the MEN scaffold, Nud1, however Kin4 interacts with Spc72 (Gryaznova et al. [2016]). To an extent, Bub2-Bfa1 may be considered an additional scaffold, as Tem1 localization is partially dependent on Bub2-Bfa1 (Caydasi et al. [2012]), as is the localization of Cdc14 (Pereira et al. [2002]). Bub2-Bfa1 is also important for localization of Cdc5 to the SPB (Botchkarev et al. [2017]). Together with Cnm67, Nud1 and Spc72 form the outer plaque of the SPB, situated on the cytoplasmic face of the structure (Fu et al. [2015]).

As FEAR release is not essential for MEN activity it has been difficult to pinpoint exactly how it contributes to mitotic exit. Cdc15 and Mob1 kinase activity are both inhibited by CDK phosphorylation (Jaspersen and Morgan [2000], König et al. [2010]), limiting their activity in metaphase, and phosphorylation of Bfa1 is important for SPoC function (Caydasi et al. [2017]). Patterns of Cdc15 and CDK localization at SPBs suggest that their SPB localization is mutually exclusive, and so FEAR activity may be required in anaphase to allow Cdc15 to interact with the SPB (König et al. [2010]). Furthermore, CDK and Cdc14 have been shown to contribute to regulation of Bfa1 by Lte1 in the absence of Kin4 (Caydasi et al. [2017]).

Scope of the model

Our proposed model aims to represent the above aspects of regulation of Cdc14 localization from metaphase to late anaphase. The roles of the MEN in cytokinesis and spindle positioning are considered outside of this scope, as is the execution of mitotic exit. In certain cases we have found differing phenotypes published for similar mutants, see for example length of mitosis in FEAR mutants (see Stegmeier et al. [2002] and Yellman and Roeder [2015]) and the role of Bmh1 in the SPoC (see Caydasi et al. [2014] and Falk et al. [2016]). In these cases we have chosen a specific account to form the basis of the model. Some mutants, such as those of *LTE1* (Low Temperature Essential), show differing phenotype at low temperature (Shirayama et al. [1994]), so our model aims to represent the cell cycle at 30°C. The impact of loss of SPoC function on cell viability is not generally detectable in yeast in lab conditions, as the efficiency of spindle alignment is so high that it will generally occur prior to cytokinesis regardless of

whether it is monitored. There are two independent spindle orientation pathways, based on either Kar9 or Dyn1, loss of either of these proteins leads to a significant delay in spindle alignment and so these mutants are used to detect SPoC defects (Scarfone and Piatti [2015]). In our model we assume that for any SPoC phenotypes, we model a cell defective in one of these pathways.

References

R. Azzam, S. L. Chen, W. Shou, A. S. Mah, G. Alexandru, K. Nasmyth, R. S. Annan, S. A. Carr, and R. J. Deshaies. Phosphorylation by cyclin B-Cdk underlies release of mitotic exit activator Cdc14 from the nucleolus. *Science*, 305(5683):516–519, July 2004.

A. J. Bardin and A. Amon. Men and sin: what's the difference? *Nature Reviews Molecular Cell Biology*, 2(11):815–826, Nov. 2001.

D. T. Bertazzi, B. Kurtulmus, and G. Pereira. The cortical protein Lte1 promotes mitotic exit by inhibiting the spindle position checkpoint kinase Kin4. *The Journal of Cell Biology*, 193(6):1033–1048, June 2011.

V. V. Botchkarev and J. E. Haber. Functions and regulation of the Polo-like kinase Cdc5 in the absence and presence of DNA damage. *Current Genetics*, 64(1):87–96, July 2017.

V. V. Botchkarev, M. V. Garabedian, B. Lemos, E. Paulissen, and J. E. Haber. The budding yeast Polo-like kinase localizes to distinct populations at centrosomes during mitosis. *Molecular Biology of the Cell*, pages mbc.E16–05–0324, Feb. 2017.

C. B. Brachmann, A. Davies, G. J. Cost, E. Caputo, J. Li, P. Hieter, and J. D. Boeke. Designer deletion strains derived from Saccharomyces cerevisiae S288C: a useful set of strains and plasmids for PCR-mediated gene disruption and other applications. *Yeast*, 14(2):115–132, Jan. 1998.

A. K. Caydasi and G. Pereira. Spindle alignment regulates the dynamic association of checkpoint proteins with yeast spindle pole bodies. *Developmental Cell*, 16(1):146–156, 2009.

A. K. Caydasi, B. Kurtulmus, M. I. L. Orrico, A. Hofmann, B. Ibrahim, and G. Pereira. Elm1 kinase activates the spindle position checkpoint kinase Kin4. *The Journal of Cell Biology*, 190(6):975–989, Sept. 2010.

A. K. Caydasi, M. Lohel, G. Grünert, P. Dittrich, G. Pereira, and B. Ibrahim. A dynamical model of the spindle position checkpoint. *Molecular Systems Biology*, 8:582, May 2012.

A. K. Caydasi, Y. Micoogullari, and B. Kurtulmus. The 14-3-3 protein Bmh1 functions in the spindle position checkpoint by breaking Bfa1 asymmetry at yeast centrosomes. *Molecular Biology of the Cell*, 2014.

A. K. Caydasi, A. Khmelinskii, R. Duenas-Sanchez, B. Kurtulmus, M. Knop, and G. Pereira. Temporal and compartment-specific signals coordinate mitotic exit with spindle position. *Nature Communications*, 8:14129, Jan. 2017.

A. I. de los Santos-Velázquez, I. G. de Oya, J. Manzano-López, and F. Monje-Casas. Late rDNA Condensation Ensures Timely Cdc14 Release and Coordination of Mitotic Exit Signaling with Nucleolar Segregation. *Current Biology*, 27(21):3248–3263.e5, Nov. 2017.

J. E. Falk, L. Y. Chan, and A. Amon. Lte1 promotes mitotic exit by controlling the localization of the spindle position checkpoint kinase Kin4. *Proceedings of the National Academy of Sciences*, 108(31): 12584–12590, Aug. 2011.

J. E. Falk, I. W. Campbell, K. Joyce, J. Whalen, A. Seshan, and A. Amon. LTE1 promotes exit from mitosis by multiple mechanisms. *Molecular Biology of the Cell*, 27(25):3991–4001, Dec. 2016.

J. Fu, I. M. Hagan, and D. M. Glover. The Centrosome and Its Duplication Cycle. *Cold Spring Harbor Perspectives in Biology*, 7(2):a015800, Feb. 2015.

M. Geymonat, A. Spanos, P. A. Walker, L. H. Johnston, and S. G. Sedgwick. In vitro regulation of budding yeast Bfa1/Bub2 GAP activity by Cdc5. *Journal of Biological Chemistry*, 278(17):14591–14594, Apr. 2003.

M. Geymonat, A. Spanos, G. de Bettignies, and S. G. Sedgwick. Lte1 contributes to Bfa1 localization rather than stimulating nucleotide exchange by Tem1. *The Journal of Cell Biology*, 187(4):497–511, Nov. 2009.

Y. Gryaznova, A. K. Caydasi, G. Malengo, and V. Sourjik. A FRET-based study reveals site-specific regulation of spindle position checkpoint proteins at yeast centrosomes. *eLife*, 5, 2016.

W.-K. Huh, J. V. Falvo, L. C. Gerke, A. S. Carroll, R. W. Howson, J. S. Weissman, and E. K. OShea. Global analysis of protein localization in budding yeast. *Nature*, 425(6959):686–691, Oct. 2003.

S. L. Jaspersen and D. O. Morgan. Cdc14 activates cdc15 to promote mitotic exit in budding yeast. *Current Biology*, 10(10):615–618, May 2000.

S. L. Jaspersen, J. F. Charles, R. L. Tinker-Kulberg, and D. O. Morgan. A late mitotic regulatory network controlling cyclin destruction in Saccharomyces cerevisiae. *Molecular Biology of the Cell*, 9 (10):2803–2817, Oct. 1998.

C. König, H. Maekawa, and E. Schiebel. Mutual regulation of cyclin-dependent kinase and the mitotic exit network. *The Journal of Cell Biology*, 188(3):351–368, Feb. 2010.

H. Maekawa, C. Priest, J. Lechner, G. Pereira, and E. Schiebel. The yeast centrosome translates the positional information of the anaphase spindle into a cell cycle signal. *The Journal of Cell Biology*, 179(3):423–436, Nov. 2007.

A. S. Mah, J. Jang, and R. J. Deshaies. Protein kinase Cdc15 activates the Dbf2-Mob1 kinase complex. *Proceedings of the National Academy of Sciences*, 98(13):7325–7330, June 2001.

D. A. Mohl, M. J. Huddleston, T. S. Collingwood, R. S. Annan, and R. J. Deshaies. Dbf2–Mob1 drives relocalization of protein phosphatase Cdc14 to the cytoplasm during exit from mitosis. *The Journal of Cell Biology*, 184(4):527–539, Feb. 2009.

G. Pereira, T. Höfken, J. Grindlay, C. Manson, and E. Schiebel. The Bub2p spindle checkpoint links nuclear migration with mitotic exit. *Molecular Cell*, 6(1):1–10, July 2000.

G. Pereira, C. Manson, J. Grindlay, and E. Schiebel. Regulation of the Bfa1p-Bub2p complex at spindle pole bodies by the cell cycle phosphatase Cdc14p. *The Journal of Cell Biology*, 157(3):367–379, Apr. 2002.

J. Ptacek, G. Devgan, G. Michaud, H. Zhu, X. Zhu, J. Fasolo, H. Guo, G. Jona, A. Breitkreutz, R. Sopko, R. R. McCartney, M. C. Schmidt, N. Rachidi, S.-J. Lee, A. S. Mah, L. Meng, M. J. R. Stark, D. F. Stern, C. De Virgilio, M. Tyers, B. Andrews, M. Gerstein, B. Schweitzer, P. F. Predki, and M. Snyder. Global analysis of protein phosphorylation in yeast. *Nature*, 438(7068):679–684, Dec. 2005.

E. Queralt and F. Uhlmann. Separase cooperates with Zds1 and Zds2 to activate Cdc14 phosphatase in early anaphase. *The Journal of Cell Biology*, 182(5):873–883, Sept. 2008.

E. Queralt, C. Lehane, B. Novák, and F. Uhlmann. Downregulation of PP2A(Cdc55) phosphatase by separase initiates mitotic exit in budding yeast. *Cell*, 125(4):719–732, May 2006.

R. J. D. Reid, S. Gonzalez-Barrera, I. Sunjevaric, D. Alvaro, S. Ciccone, M. Wagner, and R. Rothstein. Selective ploidy ablation, a high-throughput plasmid transfer protocol, identifies new genes affecting topoisomerase I-induced DNA damage. *Genome Research*, 21(3):477–486, Mar. 2011.

J. M. Rock and A. Amon. The FEAR network. *Current Biology*, 19(23):R1063–R1068, Dec. 2009.

J. M. Rock and A. Amon. Cdc15 integrates Tem1 GTPase-mediated spatial signals with Polo kinase-mediated temporal cues to activate mitotic exit. *Genes & development*, 25(18):1943–1954, Sept. 2011.

J. M. Rock, D. Lim, L. Stach, R. W. Ogrodowicz, J. M. Keck, M. H. Jones, C. C. L. Wong, J. R. Yates, M. Winey, S. J. Smerdon, M. B. Yaffe, and A. Amon. Activation of the Yeast Hippo Pathway by Phosphorylation-Dependent Assembly of Signaling Complexes. *Science*, 340(6134):871–875, May 2013.

V. Rossio and S. Yoshida. Spatial regulation of Cdc55–PP2A by Zds1/Zds2 controls mitotic entry and mitotic exit in budding yeast. *The Journal of Cell Biology*, 193(3):445–454, May 2011.

I. Scarfone and S. Piatti. Coupling spindle position with mitotic exit in budding yeast: The multifaceted role of the small GTPase Tem1. *Small GTPases*, 6(4):196–201, Oct. 2015.

M. Shirayama, Y. Matsui, K. Tanaka, and A. Toh-E. Isolation of a cdc25 family gene, msi2/lte1, as a multicopy suppressor of ira1. *Yeast*, 10(4):451–461, 1994.

W. Shou, R. Azzam, S. L. Chen, M. J. Huddleston, C. Baskerville, H. Charbonneau, R. S. Annan, S. A. Carr, and R. J. Deshaies. Cdc5 influences phosphorylation of Net1 and disassembly of the RENT complex. *BMC molecular biology*, 3(1):3, Apr. 2002.

F. Stegmeier, R. Visintin, and A. Amon. Separase, polo kinase, the kinetochore protein Slk19, and Spo12 function in a network that controls Cdc14 localization during early anaphase. *Cell*, 108(2):207–220, Jan. 2002.

F. Stegmeier, J. Huang, R. Rahal, J. Zmolik, D. Moazed, and A. Amon. The Replication Fork Block Protein Fob1 Functions as a Negative Regulator of the FEAR Network. *Current Biology*, 14(6):467–480, Mar. 2004.

J. Stoepel, M. A. Ottey, C. Kurischko, P. Hieter, and F. C. Luca. The mitotic exit network Mob1p-Dbf2p kinase complex localizes to the nucleus and regulates passenger protein localization. *Molecular Biology of the Cell*, 16(12):5465–5479, Dec. 2005.

B. N. Tomson, R. Rahal, V. Reiser, F. Monje-Casas, K. Mekhail, D. Moazed, and A. Amon. Regulation of Spo12 Phosphorylation and Its Essential Role in the FEAR Network. *Current Biology*, 19(6):449–460, Mar. 2009.

C. M. Yellman and G. S. Roeder. Cdc14 Early Anaphase Release, FEAR, Is Limited to the Nucleus and Dispensable for Efficient Mitotic Exit. *PLoS ONE*, 10(6):e0128604–24, June 2015.
