# Supplementary material for: Unifying the mechanism of mitotic exit control in a spatiotemporal logical model
Source: PLoS Biol. 2020 Nov 12;18(11):e3000917. doi: 10.1371/journal.pbio.3000917 (PMC7685450; doi:10.1371/journal.pbio.3000917)
Supplement: Attachment — Submitted filename: ResponseLetter.pdf [file pbio.3000917.s028.pdf]

Response to Reviewers *Howell, Klemm, Thorpe and Csikász-Nagy.*
Reviewers comments are in black text, our responses are in blue text and changes to the text indicated with red text.

REVIEWS:

Reviewer #1: In the manuscript entitled "Unifying the mechanism of mitotic exit control in a spatio-temporal logical model" the authors aim to provide a full mathematical model based on a novel compartmental logical modelling framework that can represent spatial regulation of the mitotic exit network (MEN) proteins. As the authors claimed the model is able to reproduce the phenotype of around 80% of published data/mutants, indicating that despite the difficulty of the network and the lack of some mechanistic details, the model can predict pretty well mitotic exit. This allowed the authors to extract some nice conclusions like the idea that FEAR is required for the regulation of the time spent in anaphase and provide some experimental data to support this point. However, some essential points in the regulation of mitotic exit are missing:

1. The model takes into account as compartments the nucleus, cytoplasm, bud, mSPB and dSPB. I find strange that the authors do not include the nucleolus as a different spatial compartment since the Cdc14 activation depends on its release from the nucleolus to the nucleus. In order to properly model FEAR-Cdc14 release to distinguish among nucleus and nucleolus is required.

We have added a nucleolus compartment to the model containing Net1, Spo12, Fob1, Hit1, Rsa1 and Cdc14. This means the model can now explicitly model the state of Cdc14 localization from nucleolus to nucleus to cytoplasm. These modifications can be seen in Figures 1 & 2.

2. In line 276 the authors state "however the activity of Cdc5 is thought to be stable throughout late mitosis, suggesting it is not part of the temporal signal initiating FEAR release." This is incorrect. It has been demonstrated that Cdc5 activation occurs as consequence of Cdc28-Clb2 phosphorylation (Mortensen et al, 2005; Rodriguez-Rodriguez et al, 2016) and that sequential activation of Cdc5 by a second Cdk1 phosphorylation is required for Cdc5 activity in late mitosis. Moreover, it was described that Cdc5 interacts with the FEAR component, separase (Rahal and Amon, 2008) and to contribute to the FEAR-Cdc14 nucleolus release (Shou et al., 2002; Visintin et al., 2003).

We agree that Cdc5 is critical for both FEAR and MEN release. There are several proteins in the model whose activity does not change, for example Spo12, but which have a phenotype when deleted. At this point we are suggesting this is the case for Cdc5. To prevent confusion we have changed the line in question to state:

“however the activity of Cdc5 is thought to be stable throughout late mitosis.”

While we agree that CDK is essential for Cdc5 activity and therefore for regulation of late mitotic events, we cannot find convincing evidence that this phosphorylation of Cdc5 is limited to anaphase. Furthermore, CDK activity is thought to decrease from metaphase to anaphase, due to degradation of cyclins by APC-Cdc20. Although it is not strictly impossible that this phosphorylation is limited to anaphase, it would require invoking another protein, probably a phosphatase. Therefore for simplicity, we limit the temporal signal initiating anaphase to Esp1, Slk19, Zds1/2 and Cdc55.

3. On page 11, the authors indicated that "restriction of mitotic exit to anaphase posed a challenge in the model". The fact that they missed the sequential activation of Cdc5 (see point 2) as other recently published FEAR-related proteins such as Hit1 (Santos-Velazquez et al 2017) and PP2A-Cdc55 role regulating MEN proteins (see points 6-7) limited their model on this aspect.

The comment "restriction of mitotic exit to anaphase posed a challenge in the model" applies to model version 0 and was fixed in versions 1-6 of the model. We have changed the following sentence from the results section to indicate this: "In particular we found that fitting phenotypes relating to the restriction of mitotic exit to anaphase posed a challenge for Model 0 and necessitated further development of the model."

We were not aware of the role of Hit1-Rsa1, so we are thankful to the reviewer for drawing our attention to this omission. We have now included Hit1-Rsa1 in the model. This is shown in Figure 1. We have also cited Santos-Velazquez et al. [2017].

4. The authors propose that their model predict a role of Cdc5 in Cdc15 localization and Lte1 regulation of Bfa1 (as stated in the abstract and in their model). However, they do not provide any experimental data supporting these findings (although they provide some experimental data supporting other points).

Experimental verification of this is beyond the scope of the paper but Cdc5 has been shown to contribute to Cdc15 localization[1], and Lte1 has been shown to contribute to the SPoC independently of Kin4[2].

[1]Rock, J. M., & Amon, A. (2011). Cdc15 integrates Tem1 GTPase-mediated spatial signals with Polo kinase-mediated temporal cues to activate mitotic exit. Genes & Development, 25(18)

[2]Caydasi, A. K., Khmelinskii, A., Duenas-Sanchez, R., Kurtulmus, B., Knop, M., & Pereira, G. (2017). Temporal and compartment-specific signals coordinate mitotic exit with spindle position. Nature Communications, 8, 14129.

5. The model failed mostly in reproducing overexpression experiments. The authors demonstrated that Cdc5 OE is not able to rescue mob1 deleted mutant as the model predicted (contrary to the observed results in thermosensitive mutants). However, since their model predicts Cdc14 activation as an output of mitotic exit activation, they should check Cdc14 localization and release instead of rescue on

plates in the mob1 deleted mutant, since other MEN-regulated proteins could be affected in this experiment (i.e. cytokinesis regulation by MEN). In the published Cdc5 OE experiments in a dbf2-2 ts mutant, Cdc14 is released only at the nucleus (not into the cytoplasm) and failed to sustain Net1 hyperphosphorylation as MEN is not active (Rodriguez-Rodriguez et al 2016). This "partial" release and intermediate phenotype on Net1 phosphorylation are not clear to be depicted in the model.

While most of the incorrect phenotypes involved overexpression, many overexpression phenotypes were accurately predicted. We have changed the text to reflect this "*While most phenotypes involving overexpression were correctly matched (40/60), many of the phenotypes which the model predicted incorrectly relate to overexpression (20/28), especially when combined with other mutations (19/28).*"

Looking again at the Rodriguez-Rodriguez et al 2016 paper, the data appear to demonstrate that Cdc14 is fully released in the *dbf2-2*, *CDC5* OE experiment. Looking particularly at Figure 7A from that paper, they show a plate where these cells appear to grow as well as the control. This is in contrast to our spot test which shows that cells lacking Mob1 cannot grow even in the presence of ectopic Cdc5.

It would be interesting to examine the phenotype of the *mob1Δ CDC5* OE strain in more detail in future studies.

6. Model 5 include a modification of the rule for Bfa1 to ensure that it can be inhibited at SPB in metaphase. The authors stated that "in this version Cdc5 is only essential to recruit Cdc15 and not in Bfa1 inhibition". This is a bit odd since regulation of Bfa1 phosphorylation is an essential step in MEN regulation and the convergent point of many checkpoint regulations (DDC, SPOC and SAC, reviewed in Matellan and Monje-Casas 2020). Moreover, the authors propose a two-step regulation on Bfa1, Kin4 dependent and Kin4 independent. As previously mentioned, the model did not include the Bfa1 dephosphorylation by PP2A-Cdc55 (Baro et al 2013) and including this step might remove the necessity for this second step on Bfa1 regulation.

This is an interesting suggestion, but a Kin4-independent pathway is required to explain the functional SPoC in *kin4Δ spo12Δ* cells. This could not be explained by PP2A-Cdc55 regulation, as PP2A-Cdc55 does not have a localization pattern that could explain this.

Due to the comments of both reviewer 1 and reviewer 2 on the topic of the essential role of Cdc5 in the model, we decided to add a new paragraph to the discussion to address these concerns. We also added figure S7B to illustrate some effects relating to Cdc5 regulation of Cdc15.

"It is interesting to note that in this model, the essential role of Cdc5 is in the localization of Cdc15 and not in inhibition of Bfa1. Certainly, if we accept that Cdc5 is inactive in metaphase and that *CDC15-7A MOB1-2A* cells have a SPoC in metaphase, we must accept that Bfa1 can become inhibited without Cdc5. The view is supported by the findings of Rock and Amon [2011] that Mob1-Dbf2 can be

activated in the absence of Cdc5 and Tem1, when Cdc15 is artificially localized at the SPB. There is some disagreement in the literature over whether deletion of *BUB2* or *BFA1* can overcome the effects of Cdc5 inactivation. While some studies have found that the lethality of *cdc5-1* (Ro et al. [2002]) and *cdc5-10* (Pereira et al. [2005]) can be totally reversed by deletion of *BUB2* or *BFA1*, other studies show these deletions cannot fully rescue *cdc5*-2, especially at 37°C (Hu et al. [2001], Fraschini et al. [2006, Miller et al. [2009]}). It is difficult to dissect the exact roles of Cdc5 because of the many roles it plays in mitosis, meaning these different temperature sensitive alleles are probably defective in slightly different functions. However, it seems clear that Cdc5 has other essential roles than just in Bub2-Bfa1 regulation. It is also worth noting that a *bfa1-11A* allele in which Cdc5 sites were mutated did not result in a mitotic arrest (Hu et al. [2001]). The model makes a number of testable predictions relating to rescue of Cdc5 mutants (Figure S7B). The model predicts that simultaneous deletion of *CDC5* and *BUB2* or *BFA1* would be able to exit mitosis. This is because the model predicts that the hyperactive Tem1 in these strains would be able to recruit Cdc15 even in the absence of Cdc5. It also recapitulates the finding of Rock and Amon [2011] that recruitment of Cdc15 to the SPB can overcome the effects of *CDC5* disruption. Furthermore, it predicts that a *CDC15-7A cdc5Δ* strain would not only be viable but would recover function of the SPoC. It seems clear that the pleiotropic nature of Cdc5 and the variable defects of different temperature sensitive *CDC5* alleles have made it difficult to precisely unravel the contribution of Cdc5 to mitotic exit. Further experiments utilising conditional systems to deplete Cdc5 activity or phosphomimetic mutations will help to test the predictions of the model and clarify the role of polo kinase. "

We thank the reviewer for drawing our attention to the review of Matellan and Monje-Casas [2020], which is now cited in the manuscript.

7. In addition, since PP2A-Cdc55 activity is in turn control by FEAR (Esp1), this will provide an additional MEN dependent regulation by FEAR, imposing a temporal order and delaying MEN activation until anaphase. Therefore, including these regulations could help to reproduce the temporal order of the FEAR and MEN activation during mitosis.

Regulation of the MEN by PP2A-Cdc55 is certainly an area of the model that could be improved. However, as FEAR network components are non-essential, it must be the case that any Cdc55 regulation is therefore also not critical. Baro et al 2013 provide some convincing evidence for premature Cdc15 activation in metaphase in *cdc55Δ* cells however they argue Cdc14 is not released due to CDK inhibition of Mob1. It would be fascinating to see if this prediction could be verified with the Mob1-2A mutant. Integrating PP2A-Cdc55 regulation into this model is certainly an area for future improvement of the model.

Minor points.

1. The abstract could be simplified and restructured to focus in the main findings demonstrated by the authors, like the deterministic timing of anaphase imposed by FEAR.

We thank the reviewer for this suggestion, the abstract has been streamlined as suggested.

"The transition from mitosis into the first gap phase of the cell cycle in budding yeast is controlled by the Mitotic Exit Network (MEN). The network interprets spatio-temporal cues about the progression of mitosis and ensures that release of Cdc14 phosphatase occurs only after completion of key mitotic events. The MEN has been studied intensively however a unified understanding of how localization and protein activity function together as a system is lacking. In this paper we present a compartmental, logical model of the MEN that is capable of representing spatial aspects of regulation in parallel to control of enzymatic activity. We show that our model is capable of correctly predicting the phenotype of the majority of mutants we tested, including mutants that cause proteins to mislocalize. We use a continuous time implementation of the model to demonstrate that Cdc14 Early Anaphase Release (FEAR) ensures robust timing of anaphase and we verify our findings in living cells. Furthermore, we show that our model can represent measured cell-cell variation in Spindle Position Checkpoint (SPoC) mutants. This work suggests a general approach to incorporate spatial effects into logical models. We anticipate that the model itself will be an important resource to experimental researchers, providing a rigorous platform to test hypotheses about regulation of mitotic exit."

2. The authors do not make it clear what is the output of the model, Cdc14 full release and activation or mitotic exit (entering into new G1).

We have added: "Although mitotic exit is executed through the concerted effort of other proteins, such as Cdh1 and Sic1, the primary trigger for mitotic exit is release of Cdc14. The output of the model is therefore the full release of Cdc14 and we will treat this as synonymous with mitotic exit."

3. Supplementary excel S6, column w is not in English

We thank the reviewer for drawing our attention to this error, it has been amended.

4. The use of many abbreviations and acronyms in supplementary files makes them difficult to follow.

We have thoroughly reworked the supplementary files in order to make them more easily accessible. We have removed abbreviations wherever possible.

5. In Fig.6 the authors used a Cdc14-CFP strain but did not report the results in the Cdc14 release during anaphase. Including this data, together to the spindle elongation will help to see whether the model and the experimental data follow the same pattern on Cdc14 activation.

We found that the spindle disassembly assay we used was a clear metric to assay the length of anaphase. Originally we hoped to use the Cdc14-CFP marker as an additional metric. However, we found that it was difficult to define a precise point where Cdc14 had been "released". This was particularly confounded by the fact that

FEAR release occurs only in one strain and not the other. We decided to go with spindle disassembly as a more reliable marker of mitotic exit. With the benefit of hindsight we could have made this work if we had included additional markers as described in *Neurohr, G., & Mendoza, M. (2016). Cdc14 Localization as a Marker for Mitotic Exit: In Vivo Quantitative Analysis of Cdc14 Release. In F. Monje-Casas & E. Queralt (Eds.), The Mitotic Exit Network (Vol. 1505, pp. 59–67). New York, NY: Springer New York.*

As it stands we have not quantitatively analyzed the Cdc14 data, as we feel the spindle marker is sufficient. However, we have now included representative images showing the Cdc14-CFP marker in both T744 and T747 in Figure S4.

6. Line 459 misspelling of dephosphorylation

We could not find use of the word "dephosphorylation" on line 459, but we have changed "de-phosphorylates" on line 45 to "dephosphorylates".

7. I am not an expert in computational biology, and I cannot assess whether the computational methods and packages used are correct or the most adequate.

Reviewer #2 (Xiaoxue Snow Zhou and Angelika Amon, signed review): This work represents an interesting new theoretical model of mitotic exit in budding yeast and deserves publication in PLOS Biology. In what follows we make several suggestions in points the authors may want to consider before publication:

In this manuscript, Howell et al. describes a compartmental logical model of the mitotic exit network (MEN) which controls exit from mitosis in budding yeast. The MEN is a GTPase-kinases signaling cascade scaffolded onto the outer plaque of the spindle pole bodies (SPBs) to sense the spindle position during mitosis. To ensure accurate genome partitioning, the MEN integrates both the spatial cue of spindle position through the MEN GTPase Tem1 and temporal cue of cell cycle progression through the downstream MEN kinases Cdc15 and Dbf2-Mob1. In addition to protein activities, localization to the SPBs is an important aspect of regulation for MEN proteins. Although the MEN has been studied extensively, a mathematical model specific to the MEN with full spatial details of the regulatory network was missing. This manuscript addresses this gap and constructs such a model for the MEN.

The authors start out by developing the framework for constructing a compartmental logical model of the MEN. A logical model is chosen over ODE models for its simplicity and scalability for a complex network such as the MEN and its regulatory network. However, this choice constrains the representation of the system to digitalized states and Boolean algebra, and as a result it is difficult to construct/interpret the model for certain protein nodes and mutants. Another assumption that the authors make to construct their model, perhaps for simplicity, is

that MEN signaling only occurs at the SPB that enters the bud/daughter cell (dSPB). The asymmetry of the two SPBs in MEN protein localization has been documented extensively and different models have been proposed to address where (which SPB(s) or off the SPBs (the cytosol)) MEN signaling occurs. Nevertheless, both Cdc15 and Dbf2-Mob1 have been shown to localize to the mSPB (the SPB stays in the mother) in addition to dSPB in early anaphase (Molk et al. 2004, Luca et al. 2001, Campbell et al. 2020) while Tem1 only localizes to the mSPB in late anaphase depending on Cdc15. This is in direct contrast to the localization pattern presented in their model (Fig. 5E-F).

We recognize these limitations in this part of the discussion: "In other cases, the model has not been able to represent effects such as low level localization of MEN proteins in metaphase (Bardin et al. [2000], Pereira et al. [2000]) or symmetric localization of MEN proteins late in anaphase (Campbell et al. [2020])."

The authors then refine the model based on a few key observations/mutant phenotypes. First, they look at CDC15-7A and MOB1-2A which bypass CDK inhibition on Cdc15/Mob1. To explain the SPoC (spindle position checkpoint) defect of CDC15-7A, they updated the regulation of Cdc15's SPB localization to include CDK inhibition where either active Tem1 or lack of CDK inhibition is sufficient for recruitment of Cdc15 to the SPB. This model predicts that Cdc15-7A should be able to bypass a tem1D and localizes to the SPB independently of Tem1. Testing these predictions experimentally would be helpful to validate this unconventional model assumption/setup. They then introduce an anaphase phase specific component (ASC) to fully explain the metaphase behavior of CDC15-7A+MOB1-2A double mutant and later propose that the polo-like kinase Cdc5 is the ASC based on previous experimental observations. Although it is known that SPB localization of Cdc15 depends on Cdc5 in addition to Tem1, this has not been tied to the observation that Cdc5 only localizes to the outer plaque of dSPB after anaphase onset. Thus, the insight that Cdc5 is an anaphase specific regulator of Cdc15 is novel and significant. However, it is misleading to start the model without incorporating or at least mentioning the known fact that Cdc5 is required for the recruitment of Cdc15 to the SPBs.

We have changed the "Restriction of mitotic exit to anaphase" section to make this clear:
"Our original model (Model 0) employs a simple rule for Cdc15 (high) localization that depends only on Tem1 and not on other known regulators, such as CDK, Cdc14 or Cdc5."

Next, the authors continue to refine their model with phenotypes of bub2D/bfa1D and kin4D+spo12D double mutant. For bub2/bfa1D, they introduce a new node for Tem1 (Tem1_high) that can simulate the metaphase exit of bub2/bfa1D. However, this model makes a few assumptions that are not well supported by experimental observations. First, in bub2/bfa1D, Tem1 does not accumulate on SPBs to notable levels until late anaphase (Caydasi et al. 2012). It is possible that the low amount of active Tem1 (~75 molecules) at SPBs observed in bub2/bfa1D is sufficient to activate Cdc15 as assumed by Caydasi et al. and the authors here. However, this is

not clearly indicated in the text. Second, the evidence for Tem1's direct involvement in recruiting Mob1 to SPBs is weak. Tethering Cdc15 to the SPBs is sufficient to bypass Tem1 (Rock and Amon 2011) and recruit Mob1 (Rock et al. 2013), indicating that Cdc15 alone is responsible for recruiting Mob1 to the SPBs. It is possible that hyperactivated Tem1 in bub2/bfa1D can hyperactivate Cdc15 which then leads to activation of Dbf2-Mob1 in metaphase without invoking Tem1's direct role in recruiting Mob1. In fact, this is consistent with the updated condition used in model 5 for Mob1.SPB (Cdc15_high.SPB & Nud1A & Mob1.Cytoplasm & Tem1_highA). Lastly, CDK inhibition on Mob1 was shown to reduce Dbf2-Mob1's kinase activity (Konig et al. 2010) rather than localization to SPBs as assumed in the model. This is further supported by the ability of Cdc15-SPB to recruit Mob1 to SPBs at any cell cycle stages (Rock et al. 2013). This modification should not affect the model predictions for most mutants but will more closely reflect the experimental observations.

In response to the comments about Bub2-Bfa1, we have added "It is an unfortunate side effect of this modelling choice that the model suggests the level of Tem1 at the SPBs is elevated in a *bfa1Δ* or *bub2Δ* strain, whereas fluorescent measurements suggest the opposite (Caydasi et al. [2012])."

In response to the comments about Mob1-Dbf2, we accept your comments however practically speaking it would not be possible for this model to incorporate these effects. We have added "It is crucial that CDK inhibits the localization and not the activity of Mob1 in order for the hyperactive state of Tem1 to be able to overcome this inhibition in metaphase in a *bub2Δ* or *bfa1Δ* strain."

When refining the model with kin4D+spo12D double mutant which restores the SPoC defect from kin4D single mutant, they rediscover that Lte1 plays additional role other than regulating Kin4 as shown by Falk et al. 2016 and Caydasi et al. 2017. Interestingly, they attribute the additional role of Lte1 to the regulation of Bfa1 rather than Tem1 using the phenotype of GAL-KIN4. They simulate two models where Lte1 either inhibits Bfa1's activity or promotes both the activity and SPB localization of Tem1 for GAL-KIN4 (Fig. S2). The latter incorrectly predicts the mitotic exit of anaphase cells with aligned spindle in GAL-KIN4 and is thus ruled out. Have the authors considered a model where Lte1 only promotes Tem1 activity but not localization to SPB? In this model, GAL-KIN4 would in theory inhibit mitotic exit in anaphase cells with aligned spindle given the ability of Kin4 to delocalize Bfa1 and Tem1 from dSPB in this mutant.

We thank the reviewers for this keen observation. We went back and tested a version of the model where Lte1 targets Tem1 activity and not localization (Model 4b). We found that this works as well as Model 4 and have included this result in Figure S2. We have changed the results section to state:
"A version of the model (Model 4a) where Lte1 targets Tem1 activity and localization directly could not correctly represent the phenotype of *KIN4* overexpression (Figure S2). However, a version (Model 4b) where Lte1 targets Tem1 activity only is as effective at explaining these phenotypes as Model 4. Regardless of the specific rule

used, it is clear that Lte1 acts through interruption of Bub2-Bfa1-mediated inhibition of Tem1."

We have also updated the discussion:
"Our modelling suggests that Lte1 prevents inhibition of Tem1 by Bub2-Bfa1, but models targeting either Tem1 or Bfa1 were equally effective at explaining the data."

Finally, the refined model was validated with 140 mutant phenotypes and successfully predicted > 80% of the phenotypes. Using this final model, the authors make predictions for forced-localization mutants and experimentally test the effect of forced SPB localization of Tem1, Cdc15 and CDK. All three cases have been tested in literature to some degree with different methods and the results shown here are consistent with previous observations. The model successfully predicted the outcome for SPB tethered Cdc15 and CDK but not for Tem1. It is unclear to us why the model predicts mitotic exit in metaphase for TEM1-SPB since both Tem1's activity (inhibited by Bub2-Bfa1) and its downstream kinases' are restricted by high CDK activity in metaphase in the model. Could the authors provide more information to help the readers understand this more intuitively?

We address this comment in the following passage from the discussion: "The model incorrectly predicts that forcing Tem1 to the SPB can initiate mitotic exit in metaphase; as demonstrated by the experiments of both Valerio-Santiago et al. [2011] and ourselves. The *bub2Δ* or *bfa1Δ* mutations cause mitotic exit to occur in metaphase by promoting premature Tem1 loading, so exploring why this mutation but not *TEM1-SPB* can have this effect will be an interesting direction for further experimentation."

In addition to the steady state logical model, the authors have also parameterized their model to incorporate continuous timing. This allows them to simulate the effect of FEAR mutants in the timing of mitotic exit and reveals an intersecting insight that has not been explicitly characterized previously regarding the role of the FEAR network in reducing cell-cell variation of mitotic exit. This is further confirmed with experimental measurements of anaphase length for FEAR mutant spo12D. They then go on to apply this continuous model to SPoC mutants and successfully explain the variations observed for SPoC mutants based on the time it takes to exit mitosis relative to spindle alignment.
In sum, this manuscript provides a valuable framework to synthesize and test observations and models from previous literature as well as future research of the MEN and contributes a step forward for the field. We recommend publication but the authors may want to consider the points raised beforehand.

Additional comments:
1. As mentioned above, the model in Fig. 1 does not include up-to-date information on the MEN, such as regulation of Cdc15 by Cdc5 and an additional Kin4-independent role of Lte1.

Figure 1 is supposed to be a simplified description, as the caption states. A more detailed description is in the supplementary info.

2. CDC15-7A +/- MOB1-2A are SPoC defective but not 100%. Could the authors simulate these mutants in their parameterized continuous model used to simulate SPoC mutants in Fig. 7 and compare the results with experimental data (Konig et al. 2010) to validate their model?

We simulated *CDC15-7A+/- MOB1-2A* cells and compared to the findings of Falk et al. [2016] in Figure 7F. Our very basic model of spindle alignment times is only really applicable to the *kar9Δ dyn1-AID* system used by Falk et al. and will probably not be accurate for other spindle alignment mutants. Konig et al [2010] use *kar9Δ* alone and Falk et al. do not include the double *CDC15-7A MOB1-2A* mutant in their analysis so we have treated *CDC15-7A MOB1-2A* as a prediction. This is distinguished by a star in Figure 7F and stated in the caption of Figure 7: "**CDC15-7A MOB1-2A* was not included in the assays of Falk et al. [2016] and so no dotted line is included."

We discuss the finding in the results section: "The model also predicts that the SPoC competence of *CDC15-7A MOB1-2A* is comparable to *CDC15-7A* alone, which would be an interesting phenomenon to test experimentally."

3. The role of Cdc5_SPB in model 5 is unclear. In Fig.4 (model 5, which appears to be almost identical as model 3a in Fig. S2), Cdc5_SPB is required for Cdc15 and thus MEN activation. How does this requirement reconcile with the model prediction for CDC15-7A+MOB1-2A double mutant where mitotic exit occurs in metaphase (when spindle migrates into the bud) without Cdc5_SPB which only occurs after anaphase onset (as described in model 3a in Fig. S2)? Adding to the confusion, there are two different rules for Cdc15_high_SPB in model 5 described in Supplementary File 3:

a. (Nud1.SPB & Tem1_lowA & Cdc15_low.SPB & Cdc5A) | (Nud1.SPB & Tem1_highA & Cdc15_low.SPB)

b. (Nud1L.SPB & Cdc15_lowL.SPB & ! CDK_lowA.Cytoplasm & Cdc5A.SPB) | (Nud1L.SPB & Cdc15_lowL.SPB & ! CDK_lowA.Cytoplasm & Tem1_lowA.SPB)

, where (a) is for Cdc15_high.SPB in the "Localization" tab and (b) is for Cdc15_highL.SPB in "LocSpecific" tab. In (a), both Tem1_lowA and Cdc5A are required in wild-type cells for Cdc15 to localize to SPBs as described by previous experimental observations (Rock et al. 2011) while in (b) either Cdc5A.SPB or Tem1_lowA.SPB is required which would resolve the issue described above for CDC15-7A+MOB1-2A but contradicts the experimental data. In addition, it appears that inhibition by CDK_low is present in both branches of (b). Which one of the three rules was used in model 5? Based on the results of model 5 for CDC15-7A+MOB1-2A, it seems that rule (b) was likely used. If so, this is not clearly indicated in the figure and is not justified since previous data show clearly that both Cdc5 and Tem1 is required for recruitment of wild-type Cdc15. A potential experiment to resolve the issue raised here with CDC15-7A+MOB1-2A is to test whether Cdc5 is required for Cdc15-7A to localize
to SPBs.

The Localization rules and the Localization-Specific rules are slightly different and so sometimes nodes appear in both sets of rules. Cdc15 localization at the SPB can be regulated by high levels of CDK either at the SPB or in the cytoplasm. The algorithm to build the logical rules assumes all activity regulators are in the same compartment as the target node. This means it would miss the regulation of Cdc15 at the SPB by CDK in the cytoplasm if we did not include a localization-specific rule for Cdc15 at the SPB too. The actual logical rule for these nodes is an OR over both rules.

4. Similarly, the rules for Lte1 and Cdc5 in regulating Bfa1 activity are inconsistent between Fig. 4 (a, a simplified diagram without locations) and Supplementary File 3 (b):

a. (! Cdc5) | (! Cdc14 & CDK_low & ! Lte1)

b. (! Cdc5A.SPB & ! Lte1A.Cytoplasm & Bfa1_lowA.SPB) | (! Cdc14_lowA.SPB & CDK_lowA.Cytoplasm & Bfa1_lowA.SPB & ! Lte1A.Cytoplasm)

Based on the text in section 4.1.3 and results of model 5, it seems that rule (b) is implemented in model 5 which puts Lte1 in both branches and thus prioritizes Lte1 over Cdc5 and Cdc14. However, this is not clearly stated or represented in the main figure/text and is not formally justified. This model predicts that Bfa1 is inhibited as long as in the same compartment with Lte1 in all cell cycle stages, which is an interesting proposal but remains to be tested. It also appears that this rule (b) serves as the foundation for the statement "In this version of the model the essential role of Cdc5 is only in the recruitment of Cdc15 and not in Bfa1 inhibition". However, just like for Bfa1 inhibition (where Cdc5 is only required in the absence of Lte1 and Cdc14), Cdc5 is no longer essential for the recruitment of Cdc15 in model 5 either (see comment #3, where Cdc5 is only required in the absence of active Tem1).

For the first part, the explanation is similar to the last point.

Due to the comments of both reviewer 1 and reviewer 2 on the topic of the essential role of Cdc5 in the model, we decided to add a new paragraph to the discussion to address these concerns. We also added figure S7B to illustrate some effects relating to Cdc5 regulation of Cdc15.

“It is interesting to note that in this model, the essential role of Cdc5 is in the localization of Cdc15 and not in inhibition of Bfa1. Certainly, if we accept that Cdc5 is restricted to the nucleus in metaphase and that *CDC15-7A MOB1-2A* cells have a SPoC in metaphase, we must accept that Bfa1 can become inhibited without Cdc5. This view is backed up by the findings of Rock and Amon [2011] that Mob1-Dbf2 can be activated in the absence of Cdc5 and Tem1, when Cdc15 is artificially localized at the SPB. There is some disagreement in the literature over whether deletion of *BUB2* or *BFA1* can overcome the effects of Cdc5 inactivation. While some studies have found that the lethality of *cdc5-1* (Ro et al. [2002]) and *cdc5-10* (Pereira et al. [2005]) can be totally reversed by deletion of *BUB2* or *BFA1*, other studies show these deletions cannot fully rescue *cdc5-2*, especially at 37C (Hu et al. [2001], Fraschini et al. [2006, Miller et al. [2009]}). It is difficult to dissect the exact roles of Cdc5 because of the many roles it plays in mitosis, meaning these different temperature sensitive alleles are probably defective in slightly different functions.

However, it seems clear that Cdc5 has other essential roles than just in Bub2-Bfa1 regulation. It is also worth noting that a *bfa1-11A* allele in which Cdc5 sites were mutated did not result in a mitotic arrest (Hu et al. [2001]). The model makes a number of testable predictions relating to rescue of Cdc5 mutants (Figure S7B). The model predicts that simultaneous deletion of *CDC5* and *BUB2* or *BFA1* would be able to exit mitosis. This is because the model predicts that the hyperactive Tem1 in these strains would be able to recruit Cdc15 even in the absence of Cdc5. It also recapitulates the finding of Rock and Amon [2011] that recruitment of Cdc15 to the SPB can overcome the effects of *CDC5* disruption. Furthermore, it predicts that a *CDC15-7A cdc5Δ* strain would not only be viable but would recover function of the SPoC. It seems clear that the pleiotropic nature of Cdc5 and the variable defects of different temperature sensitive *CDC5* alleles have made it difficult to precisely unravel the contribution of Cdc5 to mitotic exit. Further experiments utilising conditional systems to deplete Cdc5 activity or phosphomimetic mutations will help to test the predictions of the model and clarify the role of polo kinase. "

5. Localization and activity of Kin4 in metaphase. It appears in the models, Kin4 is assumed to be active and localized to SPBs in the mother cell already in metaphase which does not match the experimental observations (Chan and Amon 2009).

Kin4 appears to load in cells arrested in metaphase with nocodazole but not in cells with short spindles, according to Chan and Amon 2009. Our model cannot distinguish these situations, both are considered to just be metaphase.

6. As mentioned earlier, the localization patterns assumed and predicted by the model do not match experimental observations for most of the proteins listed (Fig. 5E-F).
Cdc5: Cdc5's localization to dSPB in late anaphase depends on Bfa1 (Botchkarev et al 2017).
Kin4: does not localized to SPBs in metaphase (Chan and Amon 2009).
Dbf2-Mob1: localizes to both SPBs in anaphase cells with correctly aligned spindles (Luca et al 2001, Campbell et al 2020, Caydasi et al 2012).
Bub2-Bfa1: localization to SPBs is reduced in anaphase cells with mispositioned spindles (Caydasi et al 2009, 2012).
Tem1: in bub2D/bfa1D, Tem1 doesn't accumulate on SPBs to notable levels until late anaphase (after mitotic exit, Caydasi et al. 2012).
Cdc14: localizes mainly to the dSPB in anaphase, likely through Bfa1 (Pereira et al 2002).

We respond to each of these individually:

- Cdc5: We have updated the model so that Cdc5 localization at the SPB depends on Bub2-Bfa1, as described in Botchkarev et al. 2017. In Model 5 it depends on the low level of Bub2-Bfa1 which is present at both SPBs, this works effectively but does not reproduce the asymmetry that Botchkarev et al. observe. We tried a variant which localized asymmetrically as it requires the high level of Bub2-Bfa1 (model 5a) but this cannot explain the phenotype of CDC15-7A MOB1-2A cells. This is shown in Figure S7A.

- Kin4: see point 5.
- Dbf2-Mob1: Localization of Dbf2-Mob1 is important for the timing of MEN signalling but is not essential (Campbell et al. [2020]). This seems to be an inherently quantitative effect, Cdc15 and Mob1-Dbf2 load at the mSPB in order to increase the rate of Mob1-Dbf2 activation as the result of a feedback loop with Cdc14. This demonstrates one of the limitations of the logical framework, as these kinds of effects can be hard to represent in this discrete framework. Ultimately we decided to model up until the moment that commitment to mitotic exit occurs, which we believe happens when Mob1-Dbf2 loads at the dSPB but not yet the mSPB. We acknowledge this shortcoming in the discussion "In other cases, the model has not been able to represent effects such as low level localization of MEN proteins in metaphase (Bardin et al. [2000], Pereira et al. [2000]) or symmetric localization of MEN proteins late in anaphase (Campbell et al. [2020])."
- Bub2-Bfa1: This effect is represented, there is more Bub2-Bfa1 at the dSPB when the spindle is aligned.
- Tem1: This is a point where the modelling framework clearly struggles to represent the real situation. In reality, it is likely that in these strains there is a small amount of fully active Tem1 but that in effect this is more active than a larger amount of Tem1 bound to Bub2-Bfa1. We resolved this by including the two levels of Tem1 activity but it unfortunately suggests that higher level of Tem1 protein is present. We have added a brief point discussing this "It is an unfortunate side effect of this modelling choice that the model suggests the level of Tem1 at the SPBs is elevated in a *bfa1Δ* or *bub2Δ* strain, whereas fluorescent measurements suggest the opposite (Caydasi et al. [2012])."
- Cdc14: We have updated the model so that Cdc14 localization at the SPB depends on the high (asymmetric) level of Bub2-Bfa1, leading to an asymmetric distribution of Cdc14 at the SPBs.

7. The phrase "early/late anaphase" typically does not refer to "pre/post-spindle alignment" since in wild-type cells anaphase onset normally coincides with movement of a SPB into the bud (spindle alignment). Rather, it refers to two different phases of anaphase defined by the spindle length.

We have edited the text in several places to make it clear when we refer to pre/post-spindle alignment.

Minor comments:

1. It would be helpful for the readers to include Kin4's role in regulating Bfa1's SPB localization in Figure 4.

Figure 4 has been modified to include Kin4's role in Bfa1 localization.

2. What is the basis for assuming Cdc15-phosphorylated Nud1 is the receptor for CDK at SPBs? In addition, what is the basis for the assumption that MEN activity (here assumed to be active Dbf2-Mob1) excludes CDK from SPB? At mSPB where Dbf2-Mob1 and CDK are both present in anaphase, this is clearly not the case.

The basis is the findings of Konig et al [2010]. In this paper, they do not propose a factor responsible for removing CDK from the SPB so we chose Mob1-Dbf2 so that activation of the MEN at the SPB would lock out CDK there. However, we take on your point that these proteins co-localize in late anaphase. Identifying the factors that exclude CDK from the dSPB would be an interesting direction for future experimental work.

3. Line 451&452 should be Fig. 5E and F.

This has been modified in the text.

4. The Supplementary Files 3 and 5 could use some descriptions for the labels (abbreviations) to help readers understand easier.

Reviewer 1 also commented on the supplementary files and because of this we have thoroughly reworked the supplementary files in order to make them more easily accessible. We have removed abbreviations wherever possible.

5. The figure # in Supplementary Files 5 is shifted by one (i.e., Fig. 2 is actually Fig. 3).

This has been amended.

Reviewer #3 (Adrien Fauré, signed review): This is a review of the manuscript "Unifying the mechanism of mitotic exit control in a spatio-temporal logical model" by Howell and colleagues. The paper introduces a logical model of the mitotic exit network (MEN), incorporating a wealth of detail, particularly regarding spatial localization of the various proteins to the nucleus, cytoplasm, bud as well as the mother- and daughter-bound spindle pole bodies. This model alone represents a valuable contribution to the field, but the authors did not stop there and could actually check some of the models predictions, showing that Cdc5 overexpression can not rescue mob1Δ mutants, that SPO12 has a role in controlling the robustness of anaphase length, or testing the recruitment of CDC15, Tem1 or Cdc28 to the SPB, thus bringing new experimental results and valuable insight into the biology of mitotic exit in the budding yeast. Moreover, some of those experiments lead to interesting methodological discussions about the strengths and limitations of the logical framework, particularly regarding overexpression and localisation mutants and discretization. On all three aspects — review of the literature and modeling, experimental predictions and tests, methodological development, I found the paper extremely compelling.

In addition I should mention that the paper is very well written. The relevant literature on the MEN is clearly presented, the logical framework and previous models discussed in (almost?) all relevant details, model construction and refinement described step by step (with one possible oversight, see below). Figures are clear. A

very pleasant read all in all.

On the minus side, there's not much to say. My main regret is the model itself was not added as supplementary material — unless I missed it? Everything else seems to be there, including R and python scripts, there's a table with the logical rules, and all the relevant data I think, but the reader who would like to play with the model would have to copy everything in the relevant format, a tedious task that stopped me from actually checking the model myself during this review. I trust the model files will be included in the final version, alongside with model annotations etc., possibly in the BoolNet or GINsim formats used by the authors, or the SBML-qual format.
The model files can be accessed at https://github.com/RowanHowell in BoolNet, SBML-qual and MaBoSS format.

In the introduction, the authors mention the tool EpiLog and then write, "there is currently no logical formalism capable of representing intracellular spatial regulation." This is I think a bit of a stretch, as indeed, although EpiLog itself only deals with multicellular systems, it relies on previous, more general work on model composition (see Mendes et al., Bioinformatics. 2013; 29(6): 749-57). To the best my knowledge this has only been applied to multicellular systems, but it could apply just as well to intracellular compartment, and I believe it deserves to be discussed.

On a similar note — there are different, albeit related, approaches to represent a logical model as a Boolean network (section 3.5). I don't think the chosen approach should influence the results, but I would still be curious to know how the authors proceeded.

This is described in the methods section: "We represented the logical model as a Boolean network, in which each level of activity is represented as an individual node, in order to make use of computational tools designed for Boolean networks."

Finally, there's also a few minor points, missing (or sometimes extra) commas, words (l. 167, 768) or apostrophes (l. 144) here and there. Longer sentences, especially those with "however", can be a little hard to parse and could be rephrased. Some abbreviations are introduced before or without their definition (dSPB in section 3.3, the "GEF" later). The lines that refer to Fig. 8C and S6C appear redundant and it seems the two figures could be mentioned together.

We have edited the manuscript with these comments in mind.

And unless I'm mistaken, "If there are n proteins represented in the model and C compartments, the resulting compartmental logical network" should have n × C nodes, and not 2 × n × C (I mean, if there are n protein and only one compartment, there would be only n nodes, right?)

The 2nC is deliberate, each protein has a localization and an activity node, so two nodes per protein and localization.

And I think that's about all? I'm happy to recommend this paper for publication after

minor revision.

Best regards,
