# Supplementary material for: Unifying the mechanism of mitotic exit control in a spatiotemporal logical model
Source: PLoS Biol. 2020 Nov 12;18(11):e3000917. doi: 10.1371/journal.pbio.3000917 (PMC7685450; doi:10.1371/journal.pbio.3000917)
Supplement: Attachment — Submitted filename: Response to Reviewers 040920.pdf [file pbio.3000917.s029.pdf]

Response to Reviewers 04.09.20 *Howell, Klemm, Thorpe and Csikász-Nagy.*
Reviewers comments are in black text, our responses are in blue text and changes to the text indicated with red text.

Reviews

Editor's comments:
Regardless of the method selected, please ensure that you provide the individual numerical values that underlie the summary data displayed in the following figure panels as they are essential for readers to assess your analysis and to reproduce it:

Fig. 6A, D, E; Fig. 7A, C, D, E, F; Fig. S5A, B, C; Fig. S6A, B, C, D and Fig. S8A, D

In File S7 you mention that the data shown in Fig. 6 is included, but we are unsure if the data underlying the graphs shown in Fig. 6A, D and E has been included. If so, please label clearly the data, otherwise please provide it.

We have reviewed the supporting information files and where necessary provided the required data. Please note that many of the numerical values plotted in Figures 6 and 7 are the output of stochastic algorithms. We have provided *example* data in File S8 and S9, but as the algorithm is stochastic, this data may not exactly match the plotted values. The algorithms used to generate this data are also available at https://github.com/RowanHowell/CLM-Python.

Reviewer 3:
The authors have answered most of my comments — except one point, where I should have been clearer:

> This is described in the methods section: "We represented the logical model as a Boolean network, in which each level of activity is represented as an individual node, in order to make use of computational tools designed for Boolean networks."

There are many possible variations on the "each level of activity is represented as an individual node" method.

GINsim / bioLQM has one implementation: http://doc.ginsim.org/lrg-modifier-bool.html http://colomoto.org/biolqm/doc/modifier-booleanization.html

but many others are possible (see Didier et al., Mapping multivalued onto Boolean dynamics, JTB 270:177-184, 2011; and Tonello, On the conversion of multivalued to Boolean dynamics, Discrete Applied Mathematics 259:193-204, 2019)

Importantly, some of the variants considered by Didier et al. do not guaranty that non-admissible states are not reachable from admissible initial states, contrary to bioLQM's implementation, and may generate non-admissible attractors.

We have modified the statement in the materials and methods section to the following and added a citation:

We represented the logical model as a Boolean network using the Van Ham mapping [39], in which each level of activity is represented as an individual node, in order to make use of computational tools designed for Boolean networks.

[39] Van Ham, Phillipe. How to deal with variables with more than two levels. Kinetic Logic a Boolean Approach to the Analysis of Complex Regulatory Systems. 1979; 326-343

The Van Ham mapping is in line with bioLQM's implementation and avoids the eccentric behaviour considered by Didier et al. (2011).
